# CIRSE Standards of Practice for the Endovascular Treatment of Visceral and Renal Artery Aneurysms and Pseudoaneurysms

**DOI:** 10.1007/s00270-023-03620-w

**Published:** 2023-11-29

**Authors:** Michele Rossi, Miltiadis Krokidis, Elika Kashef, Bora Peynircioglu, Marcello Andrea Tipaldi

**Affiliations:** 1 Department of Surgical Medical Sciences and Translational Medicine, Sapienza University of Rome-Sant’Andrea University Hospital, Rome, Italy; 2 National and Kapodistrian University of Athens, Areteion Hospital, Athens, Greece; 3 Imperial College Healthcare NHS Trust, London, UK; 4 Department of Radiology, Hacettepe University School of Medicine, Ankara, Turkey

**Keywords:** Aneurysm, Pseudoaneurysm, Embolization, Covered stents, Coils, Flow diverters

## Abstract

**Background:**

Endovascular treatment of visceral and renal artery aneurysms and pseudoaneurysms is an effective, minimally invasive treatment that has been successfully used since the early 1990s, with refined and expanded techniques and tools currently offering excellent outcomes. Due to increased detection of such lesions in recent years, many of which are asymptomatic, revision of the indications for intervention and the correct endovascular treatment approaches has become essential.

**Purpose:**

This document will presume that the indication for treatment is clear and approved by the multidisciplinary team and will define the standards required for the performance of each intervention, as well as their relative advantages and limitations. CIRSE Standards of Practice documents are not intended to impose a standard of clinical patient care, but recommend a reasonable approach to, and best practices for, the performance of the endovascular treatment of visceral and renal artery aneurysms and pseudoaneurysms.

**Methods:**

The writing group was established by the CIRSE Standards of Practice Committee and consisted of five clinicians with internationally recognised expertise in endovascular treatments. The writing group reviewed the existing literature on visceral and renal artery aneurysms and pseudoaneurysms, performing an evidence search using PubMed to identify publications in English and relating to human subjects from 1990 to 2022. The final recommendations were formulated through consensus.

**Results:**

Endovascular treatment has an established role in the successful management of visceral and renal artery aneurysms and pseudoaneurysms, and this Standards of Practice document provides up-to-date recommendations for its safe performance.

## Introduction

The CIRSE Standards of Practice Committee convened an expert writing group, tasked with writing up-to-date recommendations on the endovascular treatment of visceral and renal artery aneurysms (VAAs) and visceral and renal artery pseudoaneurysms (VAPAs). This document is not a clinical practice guideline or a systematic review of the literature. As with all CIRSE Standards of Practice documents, this document is not intended to impose a standard of clinical patient care but recommends a reasonable approach to and best practices for endovascular treatment of VAAs and VAPAs. Institutions should regularly review their internal procedures for development and improvement, taking into account international guidance, local resources and regular internal morbidity and mortality reviews.

A summary of key recommendations on the endovascular management of visceral and renal aneurysms and pseudoaneurysms can be found in Table 1.

**Table 1:** Summary of recommendations

*Indications for endovascular treatment*
Any symptomatic VAA
Any symptomatic VAPA
Any asymptomatic VAPA
Any asymptomatic gastric, gastroepiploic, pancreaticoduodenal, gastroduodenal, mesenteric or colic VAAs
Asymptomatic splenic or renal VAAs > 2 cm especially if sacciform shaped, distally located and/or with favourable anatomy
Asymptomatic hepatic, celiac, jejunal or ileal VAAs > 2 cm
Any asymptomatic VAA with increase > 0.5 cm/year
Any asymptomatic VAA in women of child-bearing age
*Pre-treatment evaluation*
Medical history
Physical examination
Cross-sectional imaging
Coagulation status
*Equipment and devices for EVT*
Sheaths and catheters of different types and lengths
Guidewires
Microcatheters
Coils (detachable/ non-detachable)
Covered stents/ Flow diverters
Liquid embolic agents
*Follow-up*
Clinical assessment of general status and access sites before discharge
CTA or MRA at 3, 12 months and yearly

*VAA* Visceral artery aneurysms; *VAPA* Visceral artery pseudoaneurysms

## Methods

The writing group for this document, which was established by the CIRSE Standards of Practice Committee, consisted of five clinicians with internationally recognised expertise in interventional radiological treatment of visceral artery aneurysms. The writing group reviewed existing literature on VAA and VAPA endovascular treatment, performing a pragmatic evidence search using PubMed relevant publications from 1980 to 2022. The writing group formulated the recommendations by consensus.

### Background

#### Definition

Visceral and renal artery aneurysms (VAAs) are rare pathologies with an incidence of 0.01–0.2% [1]. Visceral aneurysms are those affecting the splanchnic arteries and include the celiac axis and its branches, the superior mesenteric artery (SMA), the inferior mesenteric artery (IMA) and their branches, with the splenic artery involved in up to 60% of cases, followed by the hepatic artery as the second most commonly affected artery [2] (Table 2).

**Table 2:** Overview of VAAs

Affected artery	Reported incidence [8]	Gender predominance [8]	Specific and general predisposing causes and risk factors [7]	Overall VAA reported risk of rupture [15]	VAPA incidence [12]
Splenic	60–80%	F > M	Portal hypertension, pregnancy + General *	> 3.1%	8–20%
Hepatic	20%	M > F	General *	14–80%	3.4–4%
Renal	1–10%	F = M	Fibromuscular dysplasia, General *	3–30%	26–50%
Superior Mesenteric	5–7%	M = F	General *	38–50%	3–39%
Coeliac	3– 4%	M = F	General *	10–20%	10–42%
Gastroepiploic	4–5%	M = F	Celiac stenoses, + General *	up to 75% [9]	2%
Pancreatico-duodenal / gastroduodenal	2–4%	M = F	Celiac stenoses, + General *	up to 75% [9]	13–30%
Jejunal	2–4%	M = F	General *	Up to 30%	–
Ileal				Up to 30%	
Colic				Up to 70%	

*VAA/VAPA risk factors: atherosclerosis and hypertension, infection, fibromuscular dysplasia, Kawasaki disease, Osler–Weber–Rendu disease, Ehlers-Danlos syndrome, and Marfan’s syndrome [7]

*VAAs* Visceral artery aneurysms; *F* Female; *M* Male; *VAPA* Visceral artery pseudoaneurysm

A true aneurysm is defined as a focal dilatation of an artery of more than 50% of the expected diameter for the anatomical area and patient gender, involving all three vessel layers; it may have a saccular or a fusiform shape.

A false aneurysm or pseudoaneurysm is due to a contained rupture of the artery that is lined only by adventitia or by the perivascular tissues [3].

#### Natural History and Risk of Rupture

The incidence of VAAs can be as high as 10% at autopsy [1], as most are asymptomatic. Due to the widespread use of cross-sectional imaging techniques in medicine, especially computed tomography (CT), increasing numbers are being detected.

Approximately 25% of VAAs reported in the literature present with rupture and a high mortality rate [4–6] (Table 2). This is almost certainly an over-estimate in terms of their typical presentation; however, the natural history of visceral aneurysms and their risk of rupture remain poorly defined due to the rarity of the pathology and it is difficult to analyse which factors may predispose to rupture.

The main predisposing causes or risk factors of VAAs are listed in Table 2 [7–9]. Most VAAs are slow-growing or remain stable once developed; however, pregnancy does increase the risk of rupture due to underlying hormonal changes [10, 11].

The common causes of VAPA include iatrogenic injury, pancreatitis, trauma, and vasculitis [12]. The risk of rupture of VAPAs is higher than that of true aneurysms due to the lack of the three layers of the artery wall [13].

#### Clinical Presentation

Clinical symptoms are quite rare in both VAAs and VAPAs. Abdominal pain is the most common clinical presentation and may become severe at the time of rupture, when classic symptoms of acute bleeding such as haemodynamic instability and hypovolemic shock may also occur. VAAs and VAPAs can rupture into the peritoneal cavity, retroperitoneal space, gastrointestinal tract, or biliary tract. If a hepatic artery aneurysm ruptures into the biliary tree, it may present with upper GI bleeding. A splenic or pancreatic artery aneurysm may rupture into the pancreatic duct (haemosuccus pancreaticus), also presenting with gastrointestinal bleeding.

Large VAAs (over 5 cm in diameter) may present with compression symptoms, in particular abdominal discomfort and pain, lumbar pain, jaundice, or even intermittent upper gastrointestinal bleeding [14]. VAPAs will mostly have a relevant history of the precipitating underlying pathology such as pancreatitis, infective symptomatology or trauma including iatrogenic injuries. VAAs and VAPAs are almost never palpable on clinical examination.

#### Indications for Treatment

Despite the natural history being unclear, numerous reports and data available in the literature suggest that a significate percentage of VAAs present with rupture and, therefore, the management of VAAs and VAPAs usually depends on the size and location of the aneurysm, and the estimated risk of aneurysm rupture (Table 2).

No data from randomised controlled trials exist to support treatment according to the aneurysm size or shape. Historically, the treatment threshold for VAA was 2 cm [3]. Recent Society of Vascular Surgery guidelines based on retrospective studies recommend treatment of splenic or renal artery aneurysms > 3 cm in asymptomatic patients [15]. The evidence for moving the threshold from 2 to 3 cm for splenic and renal aneurysm treatment is, however, not high and remains controversial [15–18]. Therefore, this standards of practice document supports the endovascular treatment of splenic and renal aneurysms > 2 cm, especially when they have a sacciform shape, are distally located and the vascular anatomy is favourable.

Hepatic, celiac, jejunal or ileal artery aneurysms should be treated if > 2 cm [15]. Patients with gastric or gastroepiploic, pancreaticoduodenal or gastroduodenal, mesenteric or colic artery aneurysms should be treated regardless of their size due to the associated high risk of rupture [15].

VAAs with any related symptoms or in which there is a demonstrable increase in size (usually > 0.5 cm/year) should be treated regardless of their size. Treatment of asymptomatic VAAs in women of child-bearing age should be considered, particularly if an increase in size is detected.

VAPAs should be treated promptly due to the high risk of rupture, regardless of their size.

Conservative management of small VAPAs within the parenchyma may be reasonable but strict follow-up must be performed to monitor their evolution [19].

In ruptured VAAs or VAPAs, emergency treatment is required. For non-ruptured VAAs or VAPAs that could be treated either endovascularly or surgically, the procedural risk–benefit should be carefully evaluated, taking age, comorbidities and procedural complexity into consideration. The summary of recommendations is presented in Table 1.

## Patient Preparation

### Pre-procedural Imaging

Visceral and renal artery aneurysms are usually diagnosed as incidental findings in ultrasound or cross-sectional imaging. Computed tomography angiography (CTA) is the first-choice imaging modality for planning [20]. The location and size of the aneurysm, the number and size of the feeding branches (i.e. the ‘front’ and ‘back’ door), the degree of calcification, the degree of intramural thrombus and the territory that is vascularized by the aneurysm should be delineated before treatment.

A potential imaging alternative may be magnetic resonance angiography (MRA) [21]. However, MRA cannot accurately delineate calcification or the exact amount of thrombus, is more time-consuming than CTA, and might not be available in an emergency setting. On the other hand, MRI can be useful in follow-up as it is less affected by coils artefacts.

Digital subtraction angiography (DSA) adds dynamic information that cannot be obtained with cross-sectional imaging such as the collateral or intra-aneurysm flow [22]. Cone-beam CT with 3D reconstruction may offer further information on the feeding vascular network and accurate intraprocedural guidance. In select cases, it can also guide direct puncture of targeted vessels.

### Pre-procedural Work-Up

The endovascular treatment of visceral and renal aneurysms is best performed in conjunction with the anaesthetic team, under either conscious sedation or general anaesthesia, but this can be left to the operator’s preference.

Overnight hospital stay should be arranged. Local practice may dictate that the surgical team be alerted. Written informed consent must be obtained prior to the procedure. The patient’s anticoagulation status needs to be assessed and optimized for the procedure [23]. In cases where deployment of a stent is planned, double antiplatelet therapy should be administered 5 days prior to the procedure, or a loading dose of 300 mg of clopidogrel may be required on the day of the procedure.

Prophylactic intravenous antibiotic therapy should be administered within 60 min prior to the procedure and should be continued post-operatively in the event of any ischaemic sequelae.

In case of emergency intervention for a ruptured VAA or VAPA, when the lesion is recognized in the CT suite, urgent large-bore intravenous access and general aggressive supportive measures should be commenced, while the patient is brought immediately to the angiography room. The patient may also require post-operative intensive care.

## Treatment Options

### Endovascular Treatment

To date it is widely accepted in guidelines [15] that endovascular therapy is preferable to open repair; unless the endovascular approach is unsuccessful due to an occluded or endovascularly inaccessible main feeding vessel. Surgery still remains an option when endovascular exclusion would not eliminate a symptomatic mass effect, a situation sometimes associated with giant VAAs.

### General Considerations

High-resolution dynamic CTA and multi-planar reconstructions are necessary to assess the precise anatomy of the vascular territory and to determine the most appropriate treatment strategy and the easiest vascular access route. In cases of acute angulation of the feeding vessel from the aorta, brachial or radial access and the use of longer catheters and stents should be considered.

High-quality standard angiographic equipment with high-resolution fluoroscopy and a wide assortment of dedicated materials and endovascular armamentarium for emergency treatment are mandatory for treating these patients, according to the guidelines of the European, US and Canadian IR societies (CIRSE, SIR and CIRA/CAIR) [24].

Decision-making around the approach to the intervention should be based on the necessity/willingness to preserve the parent artery; the shape, size and type of aneurysm (narrow neck or fusiform/wide neck); the tortuosity and size of the parent artery; how distally the aneurysm is located [25]; and the clinical acuity of the situation.

The aim of EVT is to exclude the VAA/VAPA from the arterial circulation. This can be achieved in two ways: occluding the pathological vessel or treating the aneurysm while preserving the flow through the pathological vessel. In most cases, covered stent placement may be considered a safe and effective treatment for aneurysms of the common hepatic artery and its branches, pseudoaneurysms of the gastroduodenal artery stump and aneurysms of the splenic and proximal superior mesenteric arteries. Pseudoaneurysms with patent inflow and outflow vessels are usually treated with sandwich technique embolization. Pseudoaneurysms without outflow vessels are treated with coil embolization and thrombin injection of the pseudoaneurysm and coil embolization of the inflow vessel.

#### Treatment of Both the Aneurysm and Pathological Vessel

This is a classic endovascular technique also known as ‘isolation technique’, ‘front and back door embolization’ or ‘sandwich technique’ in which a microcatheter is advanced distally to the VAA/VAPA. Then, the efferent vessel (or vessels), often the aneurysm itself and the afferent vessel are all coil-packed during progressive retraction of the microcatheter to exclude the aneurysm and also occlude the pathological artery [26]. This approach is generally preferred for distal fusiform aneurysms or pseudoaneurysms. The risk of distal ischaemia is mitigated by the rich collateral supply in the visceral territory and in most cases has a low clinical impact; however, this might not be the case in end-organ lesions and ideally needs to be avoided in such cases if possible. In particular, distal occlusion of the renal artery may result in either renal infarction or renin-mediated hypertension [27], while distal ischaemia of the bowel can even lead to necrosis. Coil embolization of long segments of the efferent and afferent arteries should be limited in order to avoid unnecessary occlusion of collateral vessels.

Metallic coils are the most commonly used occlusive material. They should be oversized by 10–20% of the native vessel diameter to avoid possible complications such as coil migration or incomplete vessel occlusion [28]. On the other hand, oversizing the coil’s diameter may prevent the coil from forming its shape, thus reducing its thrombogenic effect [29]. The advantage of detachable coils is the possibility to be recaptured in the event of incorrect placement before final release and the availability of longer coil lengths. Some detachable coil systems are specifically designed to anchor with one end in the vessel wall, thus functioning as a plug and further limiting the risk of distal migration [30]. However, the cost of detachable coils is significantly higher than the cost of conventional pushable coils.

When dealing with large VAA/VAPAs, the ‘isolation technique’ can sometimes be challenging because it requires selective catheterization and embolization of all efferent vessels that originate from the sac; this is of paramount importance to reduce the risk of retrograde reperfusion of the aneurysm sac. In some cases, such as in giant aneurysms, initial partial embolization of the sac helps by reducing the flow into the sac and making the outflow vessels more visible and accessible. In such cases, liquid embolic agents may be injected into the sac, as smaller efferent vessels that would be difficult to catheterize selectively, may also be occluded by the liquid agent [14]. Dedicated vascular plugs (delivered through a 6 Fr or a 7 Fr sheath, depending on the size of the device) or micro-vascular plugs (delivered through a diagnostic catheter with 0.038-inch end-hole) may be used, alone or combined with coils, to occlude the feeding artery in a ‘one step’ fashion.

Liquid embolic agents have been described in this setting and are particularly useful for reaching extremely tortuous and distal small arteries (for example, the pancreatic-duodenal arcade or splenic territories) that are unsuitable for coil embolization [31], and/or in emergency cases when rapid embolization is needed, such as for traumatic bleeding in the liver and spleen. N-Butyl cyanoacrylate glue (n-BCA) has been mainly described in the treatment of pseudoaneurysms and less frequently for the treatment of VAAs [32]. It can be used for a variation of the ‘front and back door’ technique using n-BCA to fill the aneurysm sac and both the efferent and afferent vessels, with or without coiling. Ethylene vinyl alcohol copolymer (EVOH) agents can be used to treat VAPAs or VAAs together with coils to enhance their occlusion capacity [33]. EVOH’s non-adhesive properties, high radiopacity and longer solidification time offer advantages when compared to n-BCA and make the embolization procedure more controllable and predictable. Disadvantages include the cost, the required ‘activation time’ and a burning sensation for the patient due to the exothermic reaction [34].

#### Aneurysm Exclusion with Parent Vessel Flow Preservation

The aim of this endovascular treatment is to exclude the sac from the circulation while preserving the patency of the feeding vessel. The concept of vessel patency is of the utmost importance, particularly in neurointerventions. In visceral aneurysms, distal flow is crucial when dealing with vessels such as the mesenteric and the renal branches where distal ischaemia can lead to major complications, but it is nowadays considered reasonable in other vascular territories too in order to avoid post-embolization syndrome and ischaemic complications.

The most common ‘exclusion techniques’ are the following:

- *Coil packing:* this technique is often ideal in instances of saccular true aneurysms with a narrow neck. It consists of filling the sac with coils up to the neck of the aneurysm. Detachable coils are recommended as their deployment is controlled and they can be retrieved if needed. Tight coil packing is key to this procedure to minimise the risk of subsequent coil compaction and/or sac reperfusion [35]. 3D shape memory coils with a diameter corresponding to the largest measure of the sac are usually placed first, followed by smaller coils. Coil size is decided based on the size of the aneurysm. It has been shown in prospective studies that complex-shaped platinum coils facilitate higher packing density, which improved the recanalization rate, compared to the use of helical coils [36].
- *Balloon remodelling technique:* this involves the placement of a balloon catheter in the parent vessel across the aneurysm neck and a microcatheter placed into the sac. The balloon is inflated, while the coils are inserted to prevent their prolapsing into the parent vessel lumen and intermittently deflated for temporary flow recovery and to verify the coil position. It should be considered when the aneurysm’s neck is wide but still sufficient to retain the coil cast. The aim is to allow the initial formation of a ‘cage’ preventing coil prolapse. Balloon-assisted embolization can even be performed with a liquid embolic such as Onyx or EVOH [37].
- *Stent-assisted coil embolization:* when the neck of an aneurysm is too wide to be treated with a balloon remodelling technique, stent-assisted coil embolization should be considered as an alternative means of avoiding coil prolapse or migration. It consists of the deployment of an uncovered stent in the parent vessel across the aneurysm neck and a microcatheter placed into the sac alongside the stent or through its mesh. Stent-assisted coil embolization is often used in the treatment of renal artery aneurysms. In the treatment of a bifurcation aneurysm of the renal artery, two stents can be placed with a Y-shaped configuration to maintain patency of the parent artery and the branches [38].
- *Covered stent or stent-graft:* this is a valid option for excluding VAAs and preserving the distal flow either in elective or emergency cases [39–41]. Stents are preferably used in ≥ 4 mm vessels, and, due to their variable relative stiffness, in straight vessel segments. Looking at the choice of length, adequate distal and proximal landing zones (1–2 cm) are needed for effective sealing. The vascular territories for this technique are mostly the splenic, hepatic and renal artery. Venturini et al. reported in a large series of 100 patients that stent-grafts could be implanted in only 30% of cases [42]. Side branches originating from the aneurysm should be preliminarily embolized to avoid retrograde reperfusion. Double antiplatelet therapy should be performed 1–6 months after the procedure. However, occlusion of the stent does not usually induce clinically evident ischaemia, due to progressive collateral development. Recently, long-term stent occlusion (2–16%) and subsequent extravascular migration (2–8%) have been reported, especially in pseudoaneurysms, probably due to local inflammation [40, 42–44]. Antibiotic prophylaxis should be carried out to prevent the risk of stent-graft infection [41].
- *Flow-diverter stents:* have been recently introduced for the treatment of VAAs. The concept behind the flow-diverter is the capacity of the device’s tight micromesh design to induce flow ‘diversion’ through the parent vessel and sac thrombosis, preserving patency of its efferent branches, which is crucial in territories with a high risk of distal ischaemia. Flow diverters are more flexible than stent-grafts as they were initially designed for intracranial use. The main limitations of these devices are the fact that for the moment they only are available up to 8 mm in diameter and their cost is still high.

#### Direct Percutaneous Puncture Treatment

Percutaneous treatment under imaging guidance for VAAs and VAPAs has also been described in small series [45]. The percutaneous technique consists of an ultrasound (US)- or CT-guided fine needle (21–22G) puncture of the sac and subsequent embolization using thrombin, glue or EVOH under fluoroscopic real-time imaging control. The percutaneous approach may be performed in combination with an endovascular approach,s or in cases of endovascular treatment failure or only partial success [46].

Criteria reported to be favourable for percutaneous treatment are narrow-neck VAA/VAPAs and favourable anatomical location for percutaneous needle access [47].

## Post-Procedural Care and Follow-Up

Immediately after the procedure, final angiography is performed to check the complete exclusion of the aneurysm. Monitoring of the patient’s vital signs and access site haemostasis should be performed with regular checks in the recovery room.

When a direct puncture of the sac is performed, alternative early post-procedural measures such as ultrasound or CT angiography can be performed [48]. CT imaging may be preferred to exclude bleeding complications.

In cases where the procedure involved treatment of a ruptured aneurysm or where access site-related bleeding risks are high, regular full blood-count checks and additional patient monitoring should be included in early post-procedural care.

### Medication

If there is no parent artery stenting, no anticoagulation is needed, except for some special considerations such as aneurysmal sac compression of the parent artery or where coils have prolapsed into the parent artery as a technical complication. If covered or flow-diverter stenting was performed for VAA treatment, dual antiplatelet therapy (usually with 75 mg clopidogrel and 100 or 300 mg aspirin) is advised for 6 months followed by life-long monotherapy with aspirin (100 or 300 mg per day) [39, 42, 49, 50].

In emergency cases of ruptured VAAs or VAPAs, the best possible EVT strategies must be carefully considered, given that stent placement would require immediate full anticoagulation and antiplatelet therapy to guarantee stent patency, while ‘front and back door’ embolization would increase the risk of ischaemic complications.

Routine antibiotic prophylaxis (1–2 g cefazolin, 760 mg – 1.6 g cefuroxime sodium) is recommended per guidelines for arterial stent-grafting [51]. Heparin (3000–5000 IU) may also be administered after stent-graft deployment in elective cases.

### Follow-Up

No standardised follow-up protocol can be recommended after aneurysm treatment; however, peri-procedural, 3-month, 12-month, and then yearly follow-up imaging seems reasonable. Early follow-up within the first four weeks should focus on the complete occlusion of the aneurysm and the healthy perfusion of the adjacent organs.

Secondary endovascular procedures may be required in cases of persistent aneurysmal perfusion or sac regrowth, depending on the underlying cause [1, 52]. For example, coil compaction would require new coil packing; a type I endoleak after endografting may require angioplasty or relining to prolong the proximal/distal landing zone or embolization of the sac; a type II endoleak may require a collateral or direct puncture embolization.

CT angiography is the modality of choice for follow-up imaging in many published series [1]. Depending on the EVT used, CTA can be limited by artefacts from the devices used, in which case, MRA or US may also be used.

## Outcomes

In the management of symptomatic, large (> 2–3 cm) or ruptured visceral artery aneurysms, both endovascular therapy (EVT) and surgery are effective. In the emergency setting (in ruptured VAAs or VAPAs), EVT is the treatment of choice because of its minimally invasive nature. Surgery is more complex and has a higher mortality rate in ruptured cases [53, 54]. Although the need for reintervention was reported to be higher for EVT compared to surgery, EVT was found to be associated with shorter hospital stays and lower cardiovascular complication risks compared to surgery in overall VAA and VAPA treatment [55].

Technical success is defined as exclusion of the aneurysm as evaluated on completion angiography. Clinical success represents improvement in the patient’s signs and symptoms that prompted the treatment, within 30 days [56]. Overall technical and clinical success rates range from 84.2–100% and from 80–100%, respectively [57].

The isolation or sandwich technique is reported to be feasible in 89–100% of patients with VAA and VAPA [26, 58].

Sac embolization with coil packing with or without stent/balloon assistance is well documented in VAA treatment, especially for renal artery aneurysms. It is safe and effective with a technical success rate reported to be 97.5–100% [59–61]. The use of this technique is controversial for VAPA due to the unpredictable weakness and compliance of the false wall that may lead to coils repositioning/compaction or migration.

Covered stenting or stent-grafting in VAA and VAPA treatment has a technical success rate of 84.2–100% and a clinical success rate of 84–100% in the literature [39, 42, 62, 63]. Venturini et al. [42] compared technical and clinical success rates between covered stenting and embolization and they concluded that both have similar technical success rates (97% vs. 96%, respectively) with a slightly better clinical response rate in favour of covered stents (87% vs. 81.4%). The potential drawback of covered stents includes the size and rigidity of delivery systems that preclude deployment in a tortuous and small diameter vessel, with reported 2-year patency between 60–100% [39, 40, 42]. However, with the introduction of low-profile covered stents that may be advanced over an 0.018″ wire this issue is significantly reduced.

The most recent literature regarding visceral artery aneurysm treatment with flow diverter stents reported overall technical and clinical success rates of 98.5 and 83.2%, respectively. The primary stent patency rate was 87.9%, the total aneurysmal thrombosis rate was 89.8, and 93.6% of side branches remained patent at a mean follow-up time of 14.1 months [49].

The overall complication rate of EVT in this clinical setting is 0–40% [53]. EVT-related complications include end-organ ischaemia, infection ± abscess formation, target artery dissection, non-target embolization, in-stent stenosis/stent occlusion, and access site haematoma [13, 51].

End-organ ischaemia with subsequent possible abscess (e.g. splenic abscess) or organ failure (e.g. hepatic or intestinal) may be seen more frequently with the isolation or sandwich technique [28].

Non-target embolization may be more common using coils or liquid embolic agents.

The risk of access-site haematoma may be higher with covered stents due to the requirement for larger vascular sheaths up to 8 Fr, and the concomitant need for doublet antiplatelet therapy [64]; however, this risk has been reduced significantly with the use of closure devices.

Most of the reported procedure-related complications were grade 1 or 2, based on the CIRSE Classification of Complications [65]. The 30-day mortality rate was 0–13% [56]. In cohorts that have high mortality rates, most of the cases were patients with pseudoaneurysms with multiple comorbidities and/or EVT was performed emergently for ruptured aneurysms. The mortality rate was nearly zero for patients with non-ruptured true VAA that underwent endovascular therapy.

## Abbreviations

VAA: Visceral and renal artery aneurysms
VAPA: Visceral and renal artery pseudoaneurysms
EVT: Endovascular therapy
CTA: Computed tomography angiography
MRA: Magnetic resonance angiography
FLASH: Fast low-angle shot MRI
DSA: Digital subtraction angiography
n-BCA: N-Butyl cyanoacrylate glue
EVOH: Ethylene vinyl alcohol copolymer
US: Ultrasound
SIR: The Society of Interventional Radiology (USA)
CIRSE: The Cardiovascular and Interventional Radiological Society of Europe
CIRA: Canadian Interventional Radiology Association (renamed CAIR–Canadian Association for Interventional Radiology in 2018)
IU: International units
